# Targeting GTPases in Parkinson’s disease: comparison to the historic path of kinase drug discovery and perspectives

**DOI:** 10.3389/fnmol.2014.00052

**Published:** 2014-06-05

**Authors:** Lin Hong, Larry A. Sklar

**Affiliations:** ^1^Department of Pathology, The University of New MexicoAlbuquerque, NM, USA; ^2^Center for Molecular Discovery, The University of New MexicoAlbuquerque, NM, USA; ^3^Cancer Center, The University of New MexicoAlbuquerque, NM, USA

**Keywords:** Parkinson’s, GTPase, kinase, drug, multiplex

## Abstract

Neurological diseases have placed heavy social and financial burdens on modern society. As the life expectancy of humans is extended, neurological diseases, such as Parkinson’s disease, have become increasingly common among senior populations. Although the enigmas of Parkinson’s diseases await resolution, more vivid pictures on the cause, progression, and control of the illness are emerging after years of research. On the molecular level, GTPases are implicated in the etiology of Parkinson’s disease and are rational pharmaceutical targets for their control. However, targeting individual GTPases, which belong to a superfamily of proteins containing multiple members with a conserved guanine nucleotide binding domain, has proven to be challenging. In contrast, pharmaceutical pursuit of inhibition of kinases, which constitute another superfamily of proteins with more than 500 members, has been fairly successful. We reviewed the breakthroughs in the history of kinase drug discovery to provide guidance for the GTPase field. We summarize recent progress made in the regulation of GTPase activity. We also present an efficient and cost effective approach to drug screening, which uses multiplex flow cytometry and mixture-based positional scanning libraries. These methods allow simultaneous measurements of both the activity and the selectivity of the screened library. Several GTPase activator clusters were identified which showed selectivity against different GTPase subfamilies. While the clusters need to be further deconvoluted to identify individual active compounds, the method described here and the structure information gathered create a foundation for further developments to build upon.

## INTRODUCTION

Parkinson’s disease is a degenerative disorder occurring in the central nervous system (Shulman et al., 2011). Early symptoms are mostly movement related which include shaking, rigidity and slowness. As the disease progresses, thinking and behavior problems may arise with dementia common at the advanced stage. Diagnosis is usually based on symptoms, and neuroimaging is used for confirmation (Jankovic, 2008). Parkinson’s disease is associated with loss of neurons in the substantia nigra of the midbrain and accumulation of Lewy bodies, which are aggregates of the protein α-synuclein, in the remaining neurons that generate insufficient dopamine. Parkinson’s disease affects 1% of the population above age 60 and 4% of the population over 80 (de Lau and Breteler, 2006). The disease has put a huge financial burden on society costing around 23 billion dollars in the US each year (Findley, 2007). Epidemiological studies have linked exposure to pesticides as a provocative environmental factor (Freire and Koifman, 2012). However, the detailed cause and mechanism of the disease are still ill-defined. With the accumulated genetic information available, familial causes of the disease have emerged although representing a lesser percentage (Davie, 2008).

**FIGURE 1:**
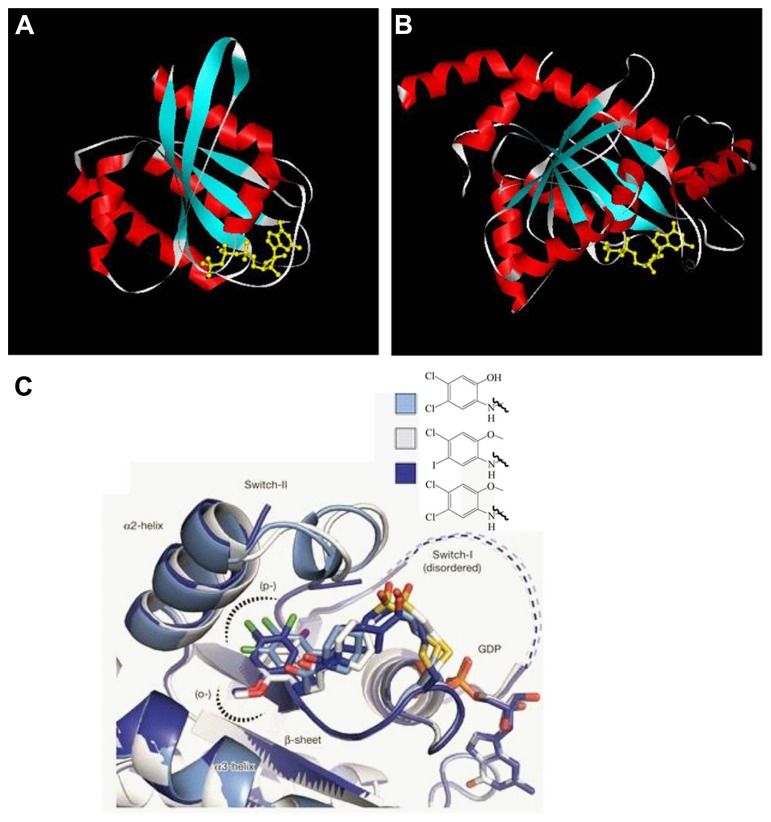
**GTPase domain core structure. (A)** Human Ras small GTPase with GTP bound. Six stranded β-sheets are surrounded by five α-helices (PDB: 121p). **(B)** *Dictyostelium* dynamin large GTPase with GDP bound. Eight stranded β-sheets are surrounded by nine α-helices (PDB: 1jwy). Helices are shown in red, β-sheets in blue, switch region in white, and the guanine nucleotide in yellow. Though the nucleotide identities are different for the two structures, the compact and globular arrangement is conserved. Both Tiff files were taken from www.endocytosis.org/Dynamin/GTPbinding-motifs.htm. **(C)** Overlaid structures of three inhibitors with GDP bound K-Ras G12C. These allosteric inhibitors bind to switch-II region and induce an inactive GDP binding conformation (Ostrem et al., 2013).

While exploring the origins of the Parkinson’s disease, proteins from various families with different functions have been suggested to contribute to its occurrence at the molecular level. One of them is the GTPase family. Multiple GTPases have been identified through genetic studies to be causal factors. GTPases are guanine nucleotide binding proteins which switch between GTP and GDP binding states. These proteins play important roles in various cellular processes, such as division, signal transduction, protein synthesis, and vesicle transport. Based on their functions, GTPases are grouped as small Ras superfamily GTPases, large GTPases, heterotrimeric G proteins, and translation factor family GTPases. In spite of size differences, these proteins have a conserved and globular guanine nucleotide binding domain which constitutes α-helixes, β-sheets, and switch I and II regions (**Figure 1**). GTPases can intrinsically hydrolyze GTP to GDP. However, the conversion between GTP and GDP is most often regulated by guanine nucleotide exchange factors (GEFs) which facilitate GDP dissociation and GTP binding, and GTPase activating proteins (GAPs) which increase the hydrolytic activity of GTPases and convert GTPases to the GDP bound state. For large GTPases, the hydrolysis of GTP powers organelle reorganization, while for small GTPases, the hydrolysis inactivates the proteins since the GDP binding conformation cannot interact with downstream effectors.

In this review, we discuss the association of GTPases with Parkinson’s disease and the potential for them to be the pharmaceutical targets. To shed light on GTPase drug discovery in more general terms, the milestones in the productive kinase field are summarized. We also describe recent progress in the search for GTPase activity regulators, including a pilot combinatorial library approach against multiple small GTPases that have been implicated in Parkinson’s disease and other disorders.

## PHYSIOLOGICAL PROCESSES ASSOCIATED WITH PARKINSON’s DISEASE AND GTPASE INVOLVEMENT

**FIGURE 2:**
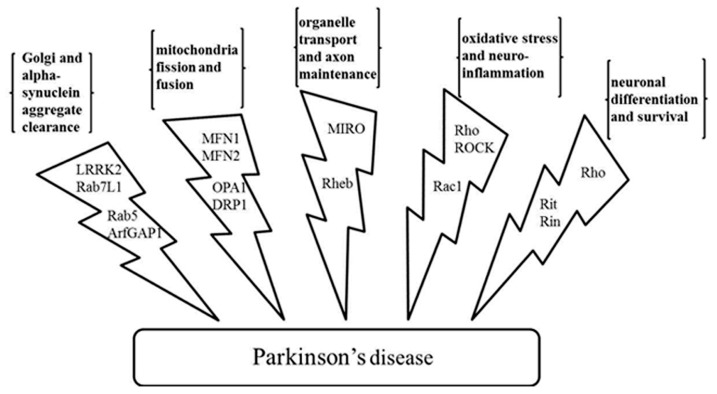
**Physiological processes related to the Parkinson’s disease and the GTPases and the effectors involved**. GTPases LRRK2, Rab7L1, Rab5, and GTPase effector ArfGAP1 regulate Golgi and α-synuclein aggregate clearance; large GTPases MFN1, MFN2, OPA1, and DRP1 regulate mitochondria fission and fusion; MIRO and Rheb have a role in organelle transport and axon maintenance; GTPase Rho, Rac1, and kinase effector ROCK are involved in oxidative stress management and neuroinflammation; Rho, Rit, and Rin are involved in neuronal differentiation and survival. LRRK2, leucine-rich repeat kinase 2; ArfGAP1, ADP-ribosylation factor GTPase-activating protein 1; MFN1 and MFN2, mitofusin-1 and mitofusin-2; OPA1, optic atrophy 1; DRP1, dynamin-related protein 1; MIRO, mitochondrial Rho-GTPase; Rheb, Ras homolog enriched in brain; ROCK, Rho-associated protein kinase.

Years of study have yielded some clues to the etiology of Parkinson’s disease. Several physiological processes are speculated to be linked to the cause and progression of the disease at the cellular level. These include organelle homeostasis and traffic, mitochondria fission and fusion, axon growth, neuron cell morphogenesis and survival, oxidative damage repair, and etc. GTPases, including large motor GTPases and small Ras superfamily GTPases, have been found to be involved in these processes. The important roles that they play are described (**Figure 2**).

### GOLGI AND α-SYNUCLEIN AGGREGATE CLEARANCE

Genetic linkage studies have linked gene *PTEN induced putative kinase 1 (PINK1)* and *PARK2* to Parkinson’s disease (Wang et al., 2011) while both the genetic linkage studies (Paisan-Ruiz et al., 2006) and genome wide association studies (GWAS; Simon-Sanchez et al., 2009) have identified leucine-rich repeat kinase 2 (LRRK2) to be genetically linked and associated with the disease. Among them, LRRK2 encodes a large multi-domain protein containing a Ras-of-complex (ROC) GTPase domain, a C-terminal of Roc (COR) domain and a serine/threonine kinase domain. The COR domain connects the GTPase and the kinase domain (Tsika and Moore, 2013). It has been suggested that the kinase and GTPase activity mutually affect each other, so that the GTP or GDP binding capacity of ROC induces kinase activation (Taymans et al., 2011) and the activated kinase phosphorylates the GTPase domain which alters conformation to further promote kinase activity (Gloeckner et al., 2010). Nonetheless, the detailed mechanism is still unresolved (Taymans, 2012). The most common mutation of LRRK2 found in Parkinson’s disease is G2019S in the kinase domain (Tsika and Moore, 2013). This mutation increases kinase activity. Another common mutation is R1441C which is in the GTPase domain (Tsika and Moore, 2013). There have been conflicting results regarding the effects of the R1441C mutation on GTP binding. However, it has been consistently demonstrated that GTPase hydrolysis activity was reduced with the mutation (Lewis et al., 2007; Daniels et al., 2011). Parkinson’s disease with mutations in the GTPase domain shows pure nigral neuron degeneration without severe Lewy body formation. Though most LRRK2 studies have been directed to control the kinase activity, it has been found that long term inhibition of LRRK kinase activity through genetic knockout has unwanted side effects including susceptibility to inflammatory bowel syndrome and kidney dysfunction (Herzig et al., 2011; Liu et al., 2011; Baptista et al., 2013). Considering the mutual regulation between the kinase and the GTPase domain, an alternative solution is to control the activity of the GTPase domain. We anticipate attempts to decrease GTP binding through minimizing the interactions between the GTPase domain and its GEFs, or efforts to increase the GTPase hydrolysis activity through controlling its GAPs could be explored (Anand and Braithwaite, 2009; Gandhi et al., 2009; Cookson, 2010; Tsika and Moore, 2013).

As for the mechanism of LRRK2 involvement in the pathogenesis of Parkinson’s disease, studies have shown that LRRK2 regulates the degradation of defective Golgi, an organelle that contributes to synaptic vesicle formation (Beilina et al., 2014). Mutations in LRRK2 therefore lead to errors in vesicular endocytosis and recycling. By screening against protein interaction arrays, LRRK2 was found to complex with several proteins (Beilina et al., 2014). The complex is important for the clearance of Golgi-derived vesicles through the autophagy-lysosome system. Both Golgi clearance and autophagy have been implicated in neuron loss in Parkinson’s disease (Fujita et al., 2006). Each protein of the complex, which is likely formed using LRRK2 as a scaffold, contributes to the Golgi clearance function. One of them, the GTPase Rab7L1 (MacLeod et al., 2013; Beilina et al., 2014), is a cytosolic GTPase belonging to the Rab family of GTPases. This GTPase has been shown to localize at the Golgi and regulate the vesicular sorting (MacLeod et al., 2013). Constitutively active Rab7L1 effectively suppressed neurite shortening induced by LRRK2 mutations. Moreover, genetic studies showed that alterations at the Rab7L1 gene promoter are likely to enhance the expression of the gene locus and are associated with reduced Parkinson’s disease risk (Gan-Or et al., 2012).

In another study, LRRK2 was also found to interact with GTPase Rab5b at the endosome in neurons which has established roles in regulating endocytosis (Shin et al., 2008). Moreover, LRRK2 interacts and phosphorylates a GAP, ADP-ribosylation factor GTPase-activating protein 1 (ArfGAP1). Silencing ArfGAP1 expression was shown to protect against mutant LRRK2 induced neurite shortening, suggesting that ArfGAP1 might also be a potential target in the Parkinson’s disease (Stafa et al., 2012; Xiong et al., 2012).

Maintaining normal homeostasis is important for cell health. Not only defective organelles but also protein aggregates need to be removed over time. Aggregates of the α-synuclein protein can form Lewy bodies that are commonly found in neurons with Parkinson’s disease. Biochemical studies have demonstrated that α-synuclein and Rab3a form a complex at the presynaptic membrane (Chen et al., 2013). Rab3a mutants with deficient GTPase activity blocked α-synuclein dissociation from the membrane. Although it is still controversial whether the sequestration of α-synuclein contributes directly to Parkinson’s disease or is associated with the disease phenotype, there appears to be a link between the intracellular level of α-synuclein and Rab3a.

Moreover, age-dependent α-synuclein accumulation could interfere with how cells cope with stress by blocking protein interactions that regulate GTPase activities. α-Synuclein in Lewy bodies was phosphorylated at Ser129 by Polo-like kinase (Plk2) both *in vitro* and *in vivo* (Wang et al., 2012a). Plk2 also interacts with and phosphorylates the GEF and/or GAPs of small GTPase Rho1. By activating downstream protein kinase C, Rho1 is an import signaling node in the mitogen-activated protein kinase (MAPK) cascade response to cell stress. When the concentration of α-synuclein is high, α-synuclein occupies Plk2 and inhibits the interaction between Plk2 and Rho1 GEFs and/GAPs. Therefore the intracellular GTP-bound and active Rho1 decreases to a level insufficient to respond to cell stress. Accumulation of such cell defects eventually lead to cell death and neuron loss is characteristic of Parkinson’s disease.

Therefore, both the cause and the consequence of α-synuclein aggregation have GTPase involvement. It is possible that by regulating GTPase activity, the formation of the α-synuclein aggregate could be reduced and its effect minimized.

### MITOCHONDRIA FISSION AND FUSION

Mitochondria are crucial for cell energy production and oxidation control. It has been recognized that the organelle undergoes dynamic fission and fusion changes. In neurons, mitochondrial fission can provide energy at sites of demand, while mitochondria fusion can regenerate mitochondrial DNA and protein after neurotoxic insult. Mounting evidence shows that dysregulated mitochondria fission and fusion contributes to Parkinson’s disease (Knott et al., 2008).

The dynamic fission and fusion of mitochondria is regulated by a group of large GTPases. The initial studies conducted in yeast have provided insight into mammalian cells (Knott et al., 2008). Mitofusin-1 and mitofusin-2 (MFN1 and MFN2) are large mammalian GTPases localized at the outer membrane of the mitochondria and direct the organelle fusion by interacting with another GTPase mitochondrial Rho-GTPase (MIRO). The amino-terminus of MFN1 and MFN2 contains the conserved GTPase domain while the carboxy-terminus contains a coiled-coil structure. Mutations in the GTPase domain of their yeast orthologs inhibit mitochondrial fusion. Also in yeast, the inner mitochondrial membrane fusion is regulated by the large GTPase Mgm1, likely through *trans* interactions between the GTPase domain and the GTPase effector domain (GED). The mammalian ortholog OPA1 may play a similar role. As for the fission of the mitochondria, large GTPase dynamin-related protein 1 (DRP1) is likely involved. Inferred from studies on the yeast ortholog dynamin 1 (Dnm1), DRP1 can form oligomers at the fission site while hydrolysis of GTP induces DRP1 to assume a conformation that helps enhances fission. Both Dnm1 and DRP1 have three conserved domains including an N-terminal GTPase, a central helical domain and a GED domain. A direct interaction between LRRK2 and DRP1 has also been demonstrated. Parkinson’s disease related mutations in the GTPase domain of LRRK2 enhanced the interaction and was shown to increase mitochondria fragmentation (Wang et al., 2012c). It has also been shown that inhibition of the large GTPase DRP1 protected neuron death both *in vitro* and *in vivo* (Grohm et al., 2012; Qi et al., 2013).

### ORGANELLE TRANSPORT ALONG AXONS AND AXON MAINTENANCE

Intracellular transport of organelles such as Golgi and mitochondria is important for maintaining healthy neurons by providing necessary substances and energy at remote synapses as well as clearing unwanted waste (Sheng and Cai, 2012; Millecamps and Julien, 2013). Errors in mitochondria movement have been associated with Parkinson’s disease (Liu et al., 2012). The characteristic α-synuclein aggregation of Parkinson’s disease was shown to impede the movement of organelles along the axons by directly interacting with axon motor proteins. The stalling of vesicles triggered a cascade of signals that lead to neuron death (Li et al., 2004). Several GTPases have been linked to the regulation of organelle transport. Besides its role in mitochondria fusion, GTPase MIRO and its adaptor proteins have also been shown to control the movement of the mitochondria (Fransson et al., 2003; Frederick et al., 2004). The GTPase anchors kinesin to the mitochondria surface to facilitate its traffic along the axons. The small GTPase Rab7 has also been shown to control endosomal and lysosomal retrograde transport (Millecamps and Julien, 2013).

Moreover, two genes, *PINK1* and *PARK2*, found to link to Parkinson’s disease from genetic linkage studies encode protein *PINK1* and Parkin. Mutations of the two proteins are often found in early onset familial forms of the Parkinson’s disease (Wang et al., 2011, 2012b). *PINK1* is a serine/threonine kinase while Parkin is an E3 ubiquitin ligase. The two proteins have been shown to cooperatively promote the ubiquitination and degradation of MIRO at the mitochondria outer membrane (Liu et al., 2012). The fusion and transport of mitochondria need to be checked since defective mitochondria might help to spread protein aggregates and be neurotoxic. *PINK1* and Parkin therefore serve as mitochondria quality control checkpoints. Mutations in *PINK1* and Parkin can cause aberrant mitochondrial homeostasis allowing the dysfunctional mitochondria to evade being engulfed by mitophagy and survive to be neurotoxic (Liu et al., 2012).

In Parkinson’s disease, it is suspected that the axon of the dopamine producing neuron plays a more important role than the cell body at the beginning stage, and it is the loss of the axon, rather than the cell body, that determines disease progression (Luo, 2000). The mammalian target of rapamycin (mTor) is a mediator of PI3K/Akt signaling. The PI3K-Akt-mTor pathway has been shown to participate in the neuron axon growth and maintenance by controlling axon number, branching, and growth cone dynamics (Kim et al., 2012). The GTPase Ras homolog enriched in brain (Rheb) is an upstream regulator which can activate mTor. By applying adeno-associated virus vector transduction, it was shown that the constitutively active GTPase Rheb could protect neurons in both normal adult mice and mice treated with neurotoxin through inducing axon sprouting and regrowth (Luo, 2000; Kim et al., 2012).

### OXIDATIVE STRESS AND NEUROINFLAMMATION

It has been shown that oxidative stress and neuroinflammation play a role in the pathogenesis and progression of Parkinson’s disease (Hirsch et al., 2012). Although the downstream signaling has not yet been elucidated, the Rho GTPase has been shown to upregulate the molecules that enhance inflammation and oxidative stress. When dopaminergic cells were treated with neurotoxins, Rho and Rho-associated protein kinase (ROCK) were activated, either directly or indirectly via the NADPH oxidase, and caused cell death. A ROCK inhibitor has been shown to alleviate the neurotoxin effect (Villar-Cheda et al., 2012). However, the effects of Rho GTPase might be multifaceted. In another study, Rho and ROCK were activated by a merlin-iso2-dependent complex and induced neurofilament heavy chain phosphorylation (Whitehead et al., 2009; Schulz et al., 2013). Down regulation of merlin-iso2 in an animal model caused symptoms of neurological disorder.

Reactive oxygen species (ROS) produced during neurotoxic stress could cause oxidative DNA damage and dopaminergic neuronal degeneration. NADPH oxidase 1 (Nox1) and small GTPase Rac1, an important regulator in the Nox1 system, were found to accumulate in the dopaminergic neurons of patients with the Parkinson’s disease (Choi et al., 2012). Moreover, in both cellular and animal models, the expression levels of both proteins were increased by neurotoxin treatment accompanied by the accumulation of damaged DNAs. Pharmaceutical or genetic intervention toward Nox1 or Rac1 attenuated the neurodegeneration.

### NEURONAL DIFFERENTIATION AND SURVIVAL

Rit and Rin are members of a novel branch of Ras superfamily GTPases and have been implicated in Parkinson’s disease by GWAS (Latourelle et al., 2012; Pankratz et al., 2012). Rit and Rin are involved in multiple cell survival signaling pathways including ERK/MAPK and p38/MAPK. Cell culture studies have suggested that Rit regulates neuron morphogenesis through the MEK/ERK pathway, and dendritic remodeling possibly through the p38/MAPK pathway. Rit knockout flies showed reduced resistance toward neurotoxic insults. Moreover, as regulators of the cell actin cytoskeleton, the Rho GTPases have also been shown to play important roles in neuronal morphogenesis. Mutations in the signaling pathway of Rho GTPases have been associated with neurological disorders (Luo, 2000).

## SUMMARY FOR PHARMACEUTICAL POTENTIAL

As has been discussed, multiple GTPases regulate various physiological processes and their misbehavior has been associated with the commencement and phenotype of Parkinson’s disease. These GTPases belong to either large motor GTPases, which contain GED and other domains besides the guanine nucleotide binding domain, or small Ras superfamily GTPases, which typically have the small or globular guanine nucleotide binding domain. A direct link of some of the GTPases to Parkinson’s disease has been explored by genetically controlling their expression at either the transcriptional or translational level. After many studies, the pathogenesis of the Parkinson’s disease is still poorly defined and the available treatments only slow down disease progression at best. Additional therapies are obviously needed. Considering the implications of the GTPases in the disease, these proteins are legitimate molecular targets. In addition, small molecule drugs have the potential advantage of ease of use and simplicity of pharmacokinetics. Therefore, searching for small molecule GTPases activity modulators is a reasonable approach. However, the hunt has appeared quite challenging, especially in comparison to kinases, another superfamily of proteins, members of which have also been implicated in many human diseases. As a whole, kinase drug discovery has been fairly productive. We summarize here the milestones in that process in an effort to provide guidance for the quest in the GTPase field.

## KINASE DRUG DISCOVERY RETROSPECTIVE

Kinases are superfamily proteins that catalyze the phosphorylation of substrates and are important for cell signal transduction, proliferation, differentiation, cell cycle, growth, and survival. With more than 500 kinases encoded in the human genome, literally every signal transduction circuit has to involve at least one phosphotransfer step (Zhang et al., 2009). A number of diseases including cancer, inflammation, and neurological disorders are caused by the misfiring of kinases. Kinases therefore have been drug targets with a long history. The kinases can be grouped into several subclasses (Noble et al., 2004): tyrosine kinases, serine threonine kinases, phosphatidylinositol 3′-kinases (PI3K), and cell cycle regulation kinases. Most success has been achieved with tyrosine kinases which can be further divided into receptor tyrosine kinases and non-receptor tyrosine kinases. Although catalysis by the different groups of kinases can be differentially regulated, the catalytic domains are well conserved in sequence and structure. The amino and carboxy termini form two separate lobe structures while the Mg-ATP complex sits in a deep cleft created by the bilobe.

It is often the case that activating mutations and overexpression of kinases lead to uncontrolled proliferation. Therefore kinase inhibitors are more commonly pursued than activators. Typically, kinase inhibitors are divided into several groups (Zhang et al., 2009): type I inhibitors bind to the ATP binding site and are competitive toward ATP; type II inhibitors bind both ATP binding site and a hydrophobic site created by the activation loop, usually in a DFG (a motif in the activation loop) out conformation; type III inhibitors bind to allosteric sites close to the ATP binding site; type IV inhibitors covalently and irreversibly bind to the active site cysteine residue of kinases.

From initial natural products that were found to have kinase inhibition activity to the designed US Food and Drug Administration (FDA)-approved drugs, the history of kinase inhibitor development represents a productive example for pharmaceutical intervention.

Staurosporine was originally isolated from the bacterium *Streptomyces staurosporeus* in 1977 and found to have antifungal and apoptosis-inducing activity (Omura et al., 1977). Earlier studies showed that staurosporine inhibited protein kinase C and bound to the ATP binding site in a competitive mode (Okazaki et al., 1988). Staurosporine is thus a prototypical type I inhibitor. However, additional studies found that staurosporine was not selective and inhibited multiple kinases (Ruegg and Burgess, 1989). This promiscuity precluded the compound from getting into clinical trials. Nonetheless, the elucidated structure of kinase CDK2 with staurosporine bound has contributed to the understanding of the interactions between the amino acids at the ATP binding site and a competitive inhibitor. Fragments of the staurosporine scaffold also helped to construct new compound libraries. Midostaurin, a derivative of staurosporine, has been studied for treatment of acute myeloid leukemia (Fischer et al., 2010).

The first FDA-approved and the most successful kinase inhibitor to date is imatinib. The break point cluster–Abelson tyrosine kinase (BCR–ABL) oncogene is formed in patients with chronic myeloid leukemia and acute lymphoblastic leukemia by fusing the BCR gene on chromosome 22 and the ABL tyrosine kinase on chromosome 9. A compound was initially discovered by screening chemical libraries to inhibit the overactive BCR–ABL kinase (Druker et al., 1996). The lead was later modified to enhance its binding affinity and given the name imatinib which receive FDA approval in 2001 (Druker et al., 2001). Imatinib was also found to inhibit both wild type c-KIT and mutant c-KIT that is often found in gastrointestinal stromal tumor (GIST) and was approved to treat the disease (Blay, 2011). The crystal structure showed that imatinib-bound BCR–ABL assumes a DFG-out inactive conformation and therefore represents the first generation type II inhibitor.

GNF2 is an allosteric type III kinase inhibitor targeting the BCL–ABL oncogene (Adrian et al., 2006). The compound did not inhibit the full length or catalytic domain of c-ABL in biochemical assays, but was as potent as imatinib in cellular proliferation assays. The crystal structure and molecule modeling showed that GNF2 bound to the myristate binding site of BCL–ABL. Therefore the compound inhibits the oncogene through a non-ATP competitive mechanism.

HKI-272 is a covalent inhibitor against epidermal growth factor receptor kinase (EGFR) which is a biomarker for many diseases including breast cancer, lung cancer, and brain tumor glioblastoma multiforme (Rabindran et al., 2004; Minami et al., 2007). HKI-272 reacts with a nucleophilic cysteine residue in EGFR through a Michael addition reaction and inhibits the autophosphorylation as well as the activation of the receptor kinase.

The above compounds represent milestones in the productive area of kinase drug discovery. By early 2009, eleven kinase inhibitors had received FDA approval for cancer treatment, and tests for treating illness other than the approved indications were ongoing in late clinical trials. At the same time, ~30 kinase targets were developed to be ready for Phase I clinical trials (Janne et al., 2009; Zhang et al., 2009). The success in kinase drug discovery undeniably surpasses what has been achieved when targeting other superfamily proteins including histone acetyltransferases and GTPases. In retrospect, the early discovery of kinase inhibitors had serendipitous elements such as finding staurosporine. As high throughput screening became available, the discovery process entered a productive phase. A number of type I ATP-competitive kinase inhibitors were identified. Lead compound optimization resulted in drugs in clinical trials, some of which were eventually approved. However, after the peak period, the movement slowed down due to the exhaustion of the available ATP-binding scaffolds. Also, the drugs obtained often lost their effectiveness due to drug resistance. Therefore, novel methods to inhibit kinases were explored. First, inhibitors with different mechanisms other than the competitive inhibition were pursued. These included type II inhibitors which could lock the kinase in an inactive conformation, type III allosteric inhibitors, and type IV covalently bound irreversible inhibitors. Drug selectivity is a common issue when the target is a member of a large superfamily of proteins. Type II and type III inhibitors are likely to have an improved selectivity profile since the binding site is not the conserved ATP binding site. As for the type IV inhibitors, although the potency has been increased, the toxicity issue still provides reservations for drug developers. Moreover, as more crystal structures of kinases with inhibitors bound are resolved, kinase drug discovery has increasingly relied on structure-based rational design. This includes lead optimization which improves upon the existing compound, structure-based design which generates novel compounds based on the knowledge obtained from the available structures, and fragment-based design where discrete fragments that bind to different parts of a target are combined to generate a new compound intended to have improved potency and selectivity. Notably, compound libraries generated by combinatorial chemical synthesis have facilitated the discovery of new kinase inhibitors where the library members can be individual compounds or compound mixtures (Liu and Gray, 2006).

## STATUS OF GTPASE DRUG DISCOVERY AND PERSPECTIVES

Like kinases, GTPases are not only implicated in neurological disorders, they have also been associated with various other human diseases including cancer and inflammation (Luo, 2000; Shaw and Cantley, 2006; Tybulewicz and Henderson, 2009). Therefore, GTPases as drug targets have been studied for several decades. Initial studies discovered GTPase regulators that obstructed GTPase membrane localization and subsequent activation through essentially inhibiting the lipid transferases that prenylate or geranylate the GTPases. However, the unselective inhibition of the lipid transferases caused severe toxicity (Konstantinopoulos et al., 2007). Compared with the success achieved in the field of kinase drug discovery, there have not yet been drugs directly targeting GTPases proceeding into late clinical trials. The scenario is likely caused by several factors. First, the GTP binding domain of GTPases is relatively small and assumes a smooth and globular structure (Milburn et al., 1990; Niemann et al., 2001; Golen, 2010). This makes it difficult to find a drug binding pocket. Second, the binding affinity of the guanine nucleotide toward the GTPases is high making other molecules difficult to compete against (John et al., 1990; Bourne et al., 1991). Third, there have been multiple established biochemical and cellular assay methods, and animal models for kinase inhibitor discovery and characterization. However, these have been relatively sparse for GTPases (Milligan, 2003; Labrecque et al., 2009). Moreover, testing GTPase activity *in vitro* normally requires high nanomolar to low micromolar enzymes, while kinase biochemical assays usually only need low nanomolar enzymes due to their high enzymatic activity (Taymans, 2012). Fourth, the activity of the GTPases is regulated by separate proteins like GEF and GAPs, instead of other domains in the same protein as in the case of kinases (Vigil et al., 2010). Finally, in comparison to kinases whose functions are mainly involved in signal transduction, GTPases appear to play more diverse roles in cell physiology ranging from cytoskeletal changes to protein translation. Therefore, toxicity from unwanted side effects can be severe (de Boer et al., 2007). Also, while overactive kinases may be problematic, both overactive and deficient GTPases have been implicated in human diseases (Sahai and Marshall, 2002). Therefore, both GTPase inhibitors and activators should be considered for different circumstances. The differences in the drug discovery process targeting kinases and GTPases are summarized in **Table 1**.

**Table 1:** Comparison of the drug discovery process between kinases and GTPases.

	Kinase	GTPase
Nucleotide binding domain structure	Potential binding pockets	Smooth and globular
Nucleotide dissociation constant	Nanomolar to micromolar	Picomolar to nanomolar
Biochemical and cellular assay availability	Ample	In development
Protein concentration in biochemical assays	Low nanomolar	High nanomolar to low micromolar
Nucleotide binding and hydrolysis regulation	Different domains on the same protein	Separate GEF and GAPs
Function range	Mostly signal transduction	Including signal transduction, cytoskeleton organization
		macromolecule transport
Misregulation	Hyperactive	Hyperactive and hypoactive

Nonetheless, powered by accumulating structural knowledge and the lessons learned from kinase drug discovery, much progress has been made in the GTPase field in recent years. This has been demonstrated in several ways. First, structure-based rational design and *in silico* screening have provided significant momentum in small molecule discovery and development (Gao et al., 2004; Shima et al., 2013). Second, in analogy to the search for the type II and type III inhibitors for the kinases, efforts have been directed to search for molecules that can either modulate the interactions between a GTPase and its effectors or that can directly inhibit the effector proteins. For example, virtual screening has identified Rho and Rac inhibitors that block the interactions between the GTPase and its GEF (Gao et al., 2004; Shang et al., 2012). From an *in silico* docking study, a Ras inhibitor was developed to inhibit the interactions between Ras and its downstream effector proteins (Shima et al., 2013). Compounds that directly inhibit the catalytic activities of the GEFs of Rho and Rac have also been developed (Nishikimi et al., 2012; Shang et al., 2013). Third, screening methods have evolved which are automated and cost efficient. A small molecule that inhibits the interactions between the farnesylated K-Ras and the prenyl-binding protein PDEδ was discovered from screening and shown to inhibit oncogenic Ras signaling (Zimmermann et al., 2013).

In our laboratory, we have developed a flow cytometry based multiplex screening and assay format (Surviladze et al., 2012) where different GTPases were linked to microsphere bead sets which had distinct fluorescence intensities and could be separated in the red fluorescence channel with excitation/emission of 635/750LP nm on a flow cytometer. The extent of fluorescent GTP binding to the individual GTPases in the presence of test compounds was analyzed by another channel with excitation/emission of 480/530 nm. This method allowed the potency and selectivity of a compound toward several GTPases to be revealed simultaneously. Also smaller quantities of GTPases were used compared to plate based homogeneous assays. Through screening the molecular library small molecule repository (MLSMR), we have identified a pan-GTPases inhibitor (Hong et al., 2010), Rho family GTPases inhibitors (Surviladze et al., 2010c, 2012), as well as a selective inhibitor for individual GTPase Cdc42 (Surviladze et al., 2010b; Hong et al., 2013). In addition, activators of GTP binding were also pursued along with the inhibitor search. Three molecules CID888706, CID7345532, and CID2160985 have been found to increase fluorescent GTP binding to multiple GTPases (Surviladze et al., 2010a). Using CID888706 as an example, biochemical studies confirmed that the compound had an EC50 in the low micromolar range and suggested an allosteric binding mechanism. Cellular assays also demonstrated that the compound could enhance the activity of Rho GTPases in regulating cytoskeleton reorganization.

**FIGURE 3:**
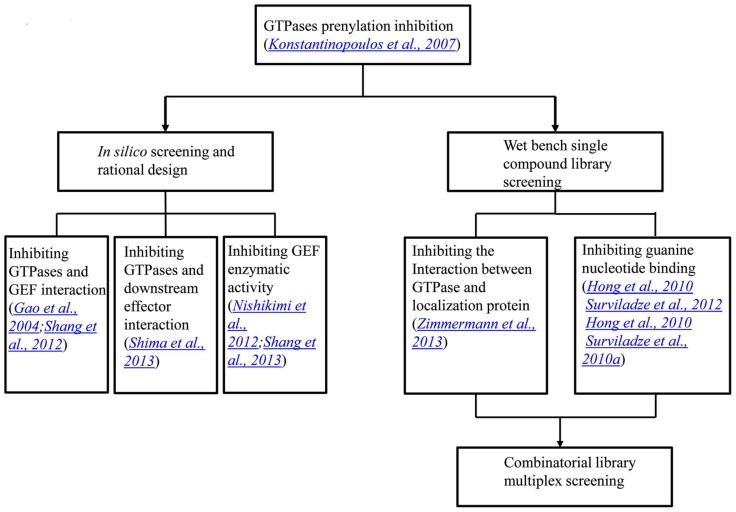
**A flow chart showing the progress in the development of GTPase activity modulators**. The initial GTPase inhibitors were natural products that inhibit GTPase prenylation and signal transduction. In the last several years, through *in silico* screening and rational design, inhibitors that blocked protein interactions and GEF enzymatic activities were identified. On the other hand, single compound library screening revealed molecules that interfere with either protein interactions or nucleotide binding. The most recent progress lies in the combinatorial library screening in a multiplex format.

We have previously reported the results of a collaborative screening effort with Torrey Pines Institute for Molecular Studies (TPIMS) involving libraries generated by combinatorial synthesis and a duplex of G protein coupled receptors (GPCRs) which resulted in a large number of the most active small molecules for the formyl peptide receptors (FPRs) ever reported (Medina-Franco et al., 2013; Pinilla et al., 2013; Santos et al., 2013). The combinatorial library contains more than 5 million small molecules and 26 million peptides. These are grouped into 37 scaffolds each of which has combinatorial derivatives of multiple functional groups (Pinilla et al., 2013). Recently, we probed this chemical library for activity modulators for GTPases in a multiplex of Rab5, Rab7, Cdc42, Ras wild type and Ras mutant Q61L with Rho and Rac screened as individual targets^1^. The GTPases used either have been associated with Parkinson’s disease or have significant roles in other illnesses. Interestingly, activators selective toward subfamilies of GTPases, including Ras and its mutant, were identified. Although the Ras inhibitors would usually be pursued, the activators of Ras can have their own uses, such as to generate cellular and animal models with aberrant Ras signaling which could be more convenient than the genetic approach. Moreover, if structures of the activator bound GTPases could be obtained, they would provide instructive information on designing GTPase activity regulators. Future collaborative studies will focus on the deconvolution of the combinatorial mixtures and the characterization of the individual or subgroups of compounds. The progress in the development of GTPase activity modulators has been recapitulated in a flow chart, as shown in **Figure 3**.

In summary, although the causes of the Parkinson’s disease have not been elucidated, the accumulating studies have shown that several cellular physiological processes are likely to be implicated in the initiation and progression of the disease, such as organelle homeostasis, axon growth and maintenance, mitochondria dynamic changes, organelle traffic, oxidative homeostasis, and neuronal cell differentiation. Enhanced by GWAS, genes implicated in the familial forms of Parkinson’s disease have been increasingly identified and their links to the disease have been studied. Multiple GTPases are involved in the disease related processes and some genes identified from GWAS encode GTPases. GTPases are therefore rational therapeutic targets for Parkinson’s disease. However, GTPase drug discovery has been progressing more slowly and only more recently when compared to its kinase counterpart. This is likely due to the fundamentally different nature of the two classes of proteins, and the different roles they have in cell physiology. Yet, lessons learned from the kinase field, such as rational design and combinatorial approaches, joined by the rapid growth of structural information, promise a leap forward in GTPase drug discovery and development in the near future.

## Conflict of Interest Statement

The authors declare that the research was conducted in the absence of any commercial or financial relationships that could be construed as a potential conflict of interest.

## Footnotes

1 The available reagents, Rho and Rac proteins, suffered from technical difficulties that created interference in the multiplex assay.
